# Method of Destroying the Fœtus in Cases of Extra-Uterine Pregnancy

**Published:** 1884-06-20

**Authors:** 


**Method of Destroying the Fœtus in Cases of Extra-Uterine Pregnancy.**—Dr .Kochmann, of Strasburg, reports a case of extra-uterine pregnancy, six months advanced, in which the fœtus was destroyed by a single application of sparks from a static battery.—*Southern Clinic.*

